# The Ireland BioClim dataset for observational and future climate projections

**DOI:** 10.1038/s41597-026-07404-y

**Published:** 2026-05-15

**Authors:** Parvaneh Nowbakht, Paraic C. Ryan, Jingyu Wang, Jenny Harmon O’Driscoll, Rosa Rogers, Mark Stewart, Enda O’Brien, Basanta Samal, Paul Nolan, Paul Holloway

**Affiliations:** 1 Department of Geography, University College Cork, Cork, Ireland; 2 Sustainability Institute, University College Cork, Cork, Ireland; 3 Discipline of Civil, Structural and Environmental Engineering, School of Engineering, University College Cork, Cork, Ireland; 4 School of English & Digital Humanities, University College Cork, Cork, Ireland; 5 School of Civil & Environmental Engineering, University of Technology Sydney, Sydney, Australia; 6 Irish Centre for High-End Computing, University of Galway, Galway, Ireland

**Keywords:** Climate sciences, Environmental sciences

## Abstract

Bioclimatic variables are essential indicators used across disciplines including ecology, geography, environmental science, and regional planning. While global datasets such as WorldClim and CHELSA provide climate information, the absence of regionally tailored datasets limits the ability to capture fine-scale climatic variability, which is critical for regional analysis. The Translate project addresses this gap by providing high-resolution (~1 km) observed and projected climate data for Ireland, enabling the generation of region-specific bioclimatic variables. The observational dataset was derived from long-term climate records using quality-controlled interpolation of national station data. An ensemble-based approach using ~200 Regional Climate Model (RCM) simulations was developed to generate percentile-based projections (10th, 50th, and 90th percentiles) across Representative Concentration Pathways (RCPs 2.6, 4.5, and 8.5) and multiple time periods. These percentiles represent a range of plausible climate futures and support uncertainty assessment. Three complementary approaches were applied: full-ensemble, period-specific, and scenario-specific outputs across RCPs and time periods. The observational and full-ensemble datasets each comprise 57 GeoTIFFs, with additional outputs provided for period- and scenario-specific analyses.

## Background & Summary

Bioclimatic variables are biologically meaningful indicators that are widely used in different areas of ecological research including assessing the impact of climate change on species^1–3^, invasion risk^4–6^, disease vectors^7–9^, forestry management^10–12^, eco-hydrology^13^, and agriculture^14–16^. The 19 bioclimatic variables, originally developed through the BIOCLIM model conceptualised by Henry A. Nix^17^ and implemented by Busby^18^, have been widely utilised within the ecological domain. A historical overview of bioclimatic variables and the BIOCLIM programme is provided by Booth^11^. These variables are arguably the most used climate dataset in ecological and environmental research^19,20^. They are derived from monthly mean, maximum, and minimum temperatures, as well as precipitation^21^, and capture essential climate patterns by representing annual trends, seasonality, and extreme environmental conditions.

Climate data, and particularly bioclimatic variables, are frequently incorporated in ecological models due to their overarching impact in determining broad scale spatial patterns^22^. WorldClim is a dependable and widely used data source that provides both baseline and future scenarios of climate^23,24^, comprising 19 bioclimatic layers in gridded format (i.e., GeoTIFF). This data is developed from various weather stations, utilizing inputs from the Global Climate Network dataset (GHCN)^25^, the World Meteorological Organization climatological database^26^, and other specialized weather station databases. These station observations are interpolated to continuous climate surfaces using spatial models and environmental predictors such as elevation and other geographic variables. Subsequent studies enhancing WorldClim have also incorporated additional covariates derived from remote sensing to improve regional interpolation and spatial representation of climate patterns^20^. With a global extent, various spatial resolutions (~30 arc-seconds [~1 km], 2.5 arc-minutes [~5 km], 5 arc-minutes [~10 km], and 10 arc-minutes [~20 km], angular units of latitude and longitude commonly used in geospatial datasets), and easy to use raster format, they are widely applicable for global, continental, regional, and local research, resulting in a strong reliance on this data by the sectors, with almost 40,000 citations at the time of writing. Despite their widespread adoption, global datasets have been subject to research that explores their accuracy at local and regional scales^20^.

Another widely used global climate dataset is CHELSA (Climatologies at High Resolution for the Earth’s Land Surface Areas)^27^. CHELSA provides high-resolution (~30 arc-second, ~1 km) global climatologies of temperature and precipitation generated by statistically downscaling atmospheric reanalysis data. In this approach, temperature fields are derived through statistical downscaling of atmospheric temperature estimates, while precipitation is modelled using orographic predictors including wind fields, valley exposure, and boundary layer height, followed by bias correction against observational station data. The CHELSA approach was originally developed using ERA-Interim reanalysis data covering approximately 1979–2013. However, the standard climatological product distributed for download represents the 1981–2010 baseline period, and the corresponding 19 bioclimatic variables are provided for this baseline climatology. Due to its explicit representation of orographic effects on precipitation, CHELSA is widely used in ecological and environmental modelling^27–29^.

Several studies have highlighted limitations in the spatial heterogeneity of climate represented in the WorldClim dataset, particularly over large and topographically complex regions^20,30,31^. While CHELSA addresses some of these limitations through improved representation of orographic effects and atmospheric processes, it is still developed as a global dataset and may therefore not fully capture fine-scale, locally specific climatic variability, particularly in regions with dense observational networks. WorldClim derives high-resolution (~1 km) climate surfaces by interpolating historical weather station data and statistically downscaling outputs from Global Climate Models (GCMs). While this approach provides accessible and consistent global coverage, it lacks the process-based representation of atmospheric dynamics that Regional Climate Models (RCMs) offer. RCMs dynamically downscale GCM output using physically based simulations at higher resolutions (typically ~12 km or finer), often requiring multiple nesting steps and significant computational resources. For instance, the Translate project, a national climate research initiative coordinated by Met Éireann and partner institutions, applies a combination of RCM-based downscaling, bias correction, detrending, and further statistical refinement to produce high-resolution (~1 km) climate projections for Ireland based on CMIP5-driven regional climate model simulations from both the EURO-CORDEX ensemble and the Nolan & Flanagan (2020) dataset^32–34^. In response to the need for localized climate information, several countries have begun to develop national climate scenarios based on outputs from the Coupled Model Intercomparison Project (CMIP)^35^. Recent research has also explored the development of regional-scale bioclimatic datasets based on ensembles of RCM simulations, including the BioVars dataset for Europe^36^, which is derived from bias-adjusted EURO-CORDEX simulations and provides bioclimatic variables across historical and future periods within a percentile-based framework. Although such datasets represent an important advancement at continental scales, their broader geographic scope, even when provided at relatively fine spatial resolution (~3 km), may still limit the representation of fine-scale, locally specific climatic variability. Some use statistical downscaling and bias correction of GCM data^37,38^, others employ dynamically downscaled RCM data to better represent local conditions^33,34,39,40^. The consensus is that where such local or regional climate models exist, they provide additional spatial, temporal, and process-level detail, such as improved representation of topographical influences, land sea contrasts, and localized weather systems, making them advantageous over GCMs^41^.

Despite the extensive use of global bioclimatic datasets in Ireland, there is currently a lack of regional-scale bioclimatic variables for Ireland. This absence is particularly concerning given the importance of such datasets for local environmental analysis and planning. However, the availability of new standardized climate data through the Met Éireann Translate project, including observational and various future Representative Concentration Pathways (RCPs) projections (2021–2050, 2041–2070, 2071–2100; RCP2.6, RCP4.5, RCP8.5) for Ireland, provides an opportunity to address this limitation. In this study, we develop a comprehensive set of 19 bioclimatic variables for the island of Ireland based on these high-resolution datasets, covering both historical and future climate conditions. To address uncertainty in future climate projections, we have adopted a percentile-based framework^42–45^.

Percentile-based methods are widely used in climate projections because they represent a range of possible outcomes, which is essential for decision-making within the context of climate uncertainty^42,45^. For instance, Böing (2010)^43^ used percentile-based rainfall scenarios to assess variability under future climate conditions, while Lutz (2016)^44^ applied percentile selection to account for performance differences in global climate models. This approach aligns with the broader recommendation in climate research to consider multiple plausible futures. Fowler and Ekström (2009)^46^ emphasized the need for multi-model ensembles and introduced a performance-based weighting approach to improve projections, while Lehner and Coats (2021)^47^ stressed the importance of considering uncertainty in climate impact assessments, particularly for long-term resilience planning. Kamruzzaman (2023)^48^ used a multi-model ensemble mean to reduce uncertainty and better understand shifts in thermal bioclimatic indicators, reinforcing the value of ensemble-based methods in capturing a broader range of possible outcomes. The ensemble-based future projections of bioclimatic variables using Translate data provide a detailed and flexible view of potential climate futures, addressing uncertainty through percentile-based scenarios.

By generating this dataset and comparing the results to the widely used WorldClim and CHELSA dataset, we aim to facilitate their application in various case studies, ultimately contributing to more effective regional climate assessments and decision-making processes. While the EPA Ireland Report No. 339^34^ presents a percentile-based approach closely aligned with the method applied in this study, other recent studies use percentiles in different contexts to summarize uncertainty in ecological forecasting and short-term rainfall risk assessment^42,43^. Although these studies are not based on multi-model climate projections, they demonstrate the broad utility of percentile methods for representing uncertainty and variability, reinforcing the value of the percentile-based framework proposed in this study. This paper will illustrate a framework to develop automated and scalable national BioClim datasets based on RCM projections, capturing additional spatial, temporal, and process-level detail, such as improved representation of topographical influences, land-sea contrasts, and localized weather systems. Implementing such an approach at national scales across Europe and worldwide would enhance the regional accuracy of projections, helping to capture important regional variability in climate impacts^49^ and improve adaptation feasibility^50^.

Although this study focuses on Ireland, the methodological framework is transferable and could be adapted to other countries or regions, provided that high-resolution regional climate data (such as those derived from RCM simulations) are available. In such contexts, the framework, methodology, and code presented here offers a flexible and scalable tool for producing localized bioclimatic datasets to support climate impact assessments and inform regional planning efforts.

## Methods

### Observational climate data

The observational dataset used in this study represents the 1976–2005 baseline period employed in the Translate climate projections. High-resolution gridded observational datasets (~1 km grid-spacing) for Ireland were obtained from Met Éireann^33,51^, including daily mean (derived from 3-hourly observations), minimum, and maximum surface air temperatures at 2 m height, as well as daily precipitation totals for the 30-year period from 1976 to 2005. The rainfall dataset is derived from a dense national network of approximately 600–700 daily rainfall stations in operation during the 1976–2005 period, while temperature data are based on 138 meteorological stations, supplemented by observations from Northern Ireland provided by the UK Met Office^52,53^. The observations underwent quality control procedures, including missing value infilling and outlier detection, and were spatially interpolated to a 1 km grid using regression-kriging^52,53^.

While the observational precipitation data covered both land and sea, temperature data was limited to land areas only. Temperature data for Northern Ireland was sourced from the UK Met Office’s CEDA archive^54^, with a 5 km grid-spacing. Bilinear interpolation was applied to seamlessly merge temperature fields across the border between the Republic of Ireland and Northern Ireland. These 30-year, quality-controlled, high-resolution datasets for daily temperature and precipitation were then used to derive 19 bioclimatic variables for Ireland, referred to here as the observational Translate BioClim dataset.

### Projections data from the translate project

The Translate climate projections consist of a collection of 30-year series of daily climate data in NetCDF format, including daily mean, minimum, and maximum temperature (at 2 m height), as well as daily precipitation. The future projections were based on CMIP5 model simulations and were dynamically and statistically downscaled to a high resolution over Ireland. Standardized climate projections for Ireland were generated from two primary sources: the higher-resolution EURO-CORDEX ensemble (~12 km grid), as used in O’Brien and Nolan (2023)^33^, and the even finer-resolution (4 km) ensemble developed by Nolan and Flanagan (2020)^34^. Although both ensembles are nested within the same CMIP5 global climate models, they differ in their RCMs, spatial resolution, and ensemble sizes. The EURO-CORDEX ensemble includes 19–29 members (depending on the RCP scenario) at a spatial resolution of 12 km, while the Nolan and Flanagan (2020)^34^ ensemble comprises 4–6 members at a finer 4 km resolution. These differences reflect trade-offs in ensemble design and computational resources: the broader EURO-CORDEX ensemble offers diversity in model responses, whereas the higher-resolution Nolan and Flanagan ensemble provides greater spatial detail over Ireland. Notably, regional climate simulations such as those in EURO-CORDEX have demonstrated added value over global models by better representing topographic and land sea effects, and by capturing higher daily precipitation intensities and more localised climate signals that are often absent in coarser global models^55^. The Nolan and Flanagan ensemble offers improved spatial precision through the use of regional model setups and observational inputs specifically calibrated for the Irish climate.

Projections spanned three future periods (2021–2050, 2041–2070, and 2071–2100) under different RCP scenarios (RCP2.6, RCP4.5, RCP8.5). See Table 1 for a list of RCMs used. Both the EURO-CORDEX and Nolan–Flanagan projection ensembles were first detrended, then bias-corrected and statistically downscaled to a common ~1 km grid, producing two separate high-resolution intermediate climate datasets. Temperature variables were detrended linearly over each 30-year daily timeseries, while daily precipitation was detrended multiplicatively, as described in O’Brien & Nolan (2023, Section 2.4.1)^33^. Variables from the Nolan & Flanagan ensemble were simultaneously bias-corrected and downscaled (from 4 km to ~1 km) using quantile delta mapping^56^, while EURO-CORDEX output was bias-corrected at its native ~12 km resolution (also using quantile delta mapping), due to storage constrains on this much larger ensemble. The EURO_CORDEX data was then statistically downscaled to ~1 km using “degraded observation” method as also described in O’Brien & Nolan (2023, Section 2.4.3)^33^, whereby the fine-scale information “lost” by interpolating observations from ~1 km grid to the coarser ~12 km grid and back again was added to the EURO_CORDEX simulation.

**Table 1:** List of EURO-CORDEX (~12 km) and Nolan & Flanagan (2020) (4 km) ensembles analysed as part of the Translate project.

EURO-CORDEX		Nolan and Flanagan (2020)	
GCM	RCM	GCM	RCM
MPI-ESM-LR	CLMcom-CCLM4 GERICS-REMO2015 KNMI-RACMO22E MPI-CSC-REMO2009 SMHI-RCA4	MPI-ESM-LR	COSMO-CLM5
CNRM-CM5	CLMcom-CCLM4 CNRM-ALADIN53 KNMI-RACMO22E RMIB-Ugent-ALARO SMHI-RCA4	CNRM-CM5	COSMO-CLM5
EC-EARTH	CLMcom-CCLM4 DMI-HIRHAM5 KNMI-RACMO22E SMHI-RCA4	EC-EARTH	COSMO-CLM5
IPSL-CM5A-MR	IPSL-INERIS-WRF331F KNMI-RACMO22E SMHI-RCA4		
NorESM1-M	DMI-HIRHAM5 GERICS-REMO2015 KNMI-RACMO22E		
HadGEM2	CLMcom-CCLM4 GERICS-REMO2015 KNMI-RACMO22E SMHI-RCA4	HadGEM2	COSMO-CLM5
		MIROC5	COSMO-CLM5 WRF

Bioclimatic variables were then generated independently from each downscaled ensemble before being combined into a single ensemble for percentile-based analysis. After harmonization to the common ~1 km grid, simulations from both ensembles were combined and treated as independent members of a pooled ensemble to represent a broader range of model responses. In this framework, percentile values reflect the empirical distribution of all model responses rather than being associated with a specific dataset, with only limited influence from ensemble composition, as differences between datasets and model-sensitivity categories are small relative to the overall variability in the combined distribution.

The projections used in this study are based on CMIP5-driven simulations, as CMIP6-based EURO-CORDEX downscaled projections are not yet fully available for Europe. While CMIP6-based regional ensembles are emerging for Ireland^40^ (e.g., Nolan, 2024), their current coverage remains limited. Previous comparisons between CMIP5 and CMIP6 simulations indicate that some quantitative differences may arise, while the overall spatial patterns and qualitative conclusions are generally consistent^57^.

### Bioclimatic variable generation

The process of generating bioclimatic variables was implemented using Python and Jupyter notebooks, with scripts developed to automate the workflow, utilizing the BIOCLIM sub-module from the Xclim package^58^ and the ANUCLIM (v6.1)^59^. The BIOCLIM module in Xclim calculates bioclimatic parameters based on temperature and precipitation data, and expects temperature inputs to be provided in Kelvin, in line with its default conventions for physical units. The output is then converted to degrees Celsius for better interpretability and familiarity. According to Xu and Hutchinson (2011)^59^, input values should ideally be provided at a weekly or monthly frequency, as bioclimatic variables primarily capture monthly, seasonal, and annual trends, and do not typically require the finer temporal resolution of daily or hourly data. Additionally, climate data at these coarser temporal resolutions are often more widely available and less computationally intensive to process, although daily data can also be used to generate bioclimatic variables such as BIO1, BIO12, and BIO10 (Table 2). For consistency, we used monthly climate data throughout the analysis. Alternatively, the biovars function from R package dismo^60^ can be used to develop equivalent scripts in R^61^.

**Table 2:** Description and formulas of the 19 bioclimatic variables, including definitions, units, and calculation methods.

Variable	Description	Unit
${\rm{BIO}}1=\frac{\mathop{\sum }\limits_{i=1}^{i=12}{{Tmean}}_{i}}{12}$	Annual Mean Temperature	°C
${\rm{BIO}}2=\frac{\mathop{\sum }\limits_{i=1}^{i=12}{(T{\max }}_{i}-{T{\min }}_{i})}{12}$	Mean Diurnal Range (Mean of monthly (max temp - min temp))	°C
${\rm{BIO}}3=\frac{{BIO}2}{{BIO}7}\times 100$	Isothermality (BIO2/BIO7) ( × 100)	%
${\rm{BIO}}4={SD}\left\{{{Tmean}}_{1},\ldots ,{{Tmean}}_{12}\right\}\times 100$	Temperature Seasonality (standard deviation × 100)	°C (SD × 100)
${\rm{BIO}}5={\max }(\left\{{T{\max }}_{1},\ldots ,{T{\max }}_{12}\right\})$	Max Temperature of Warmest Month	°C
${\rm{BIO}}6={\min }(\left\{{T{\min }}_{1},\ldots ,{T{\min }}_{12}\right\})$	Min Temperature of Coldest Month	°C
${\rm{BIO}}7={BIO}5-{BIO}6$	Temperature Annual Range (BIO5-BIO6)	°C
${\rm{BIO}}8=\frac{\mathop{\sum }\limits_{i=1}^{i=3}{{Tmean}}_{i}}{3}$ where *i* ϵ ${{WQ}}_{{PPT}}$	Mean Temperature of Wettest Quarter	°C
${\rm{BIO}}9=\frac{\mathop{\sum }\limits_{i=1}^{i=3}{{Tmean}}_{i}}{3}$ where *i* ϵ ${{DQ}}_{{PPT}}$	Mean Temperature of Driest Quarter	°C
${\rm{BIO}}10=\frac{\mathop{\sum }\limits_{i=1}^{i=3}{{Tmean}}_{i}}{3}$ where *i* ϵ ${Q}_{T{\max }}$	Mean Temperature of Warmest Quarter	°C
${\rm{BIO}}11=\frac{\mathop{\sum }\limits_{i=1}^{i=3}{{Tmean}}_{i}}{3}$ where *i* ϵ ${Q}_{T{\min }}$	Mean Temperature of Coldest Quarter	°C
${\rm{BIO}}12={\sum }_{i=1}^{i=12}{{PPT}}_{i}$	Annual Precipitation	mm
${\rm{BIO}}13={\max }(\left\{{{PPT}}_{1},\ldots ,{{PPT}}_{12}\right\})$	Precipitation of Wettest Month	mm
${\rm{BIO}}14={\min }(\left\{{{PPT}}_{1},\ldots ,{{PPT}}_{12}\right\})$	Precipitation of Driest Month	mm
${\rm{BIO}}15=\frac{{SD}\{{{PPT}}_{1},\ldots ,{{PPT}}_{12}\}}{1+(\frac{{BIO}12}{12})}\times 100$	Precipitation Seasonality (Coefficient of Variation)	%
${\rm{BIO}}16={{WQ}}_{{PPT}}$	Precipitation of Wettest Quarter	mm
${\rm{BIO}}17={{DQ}}_{{PPT}}$	Precipitation of Driest Quarter	mm
${\rm{BIO}}18=\mathop{\sum }\limits_{i=1}^{i=3}{{PPT}}_{i}$ where *i* ϵ ${Q}_{T{\max }}$	Precipitation of Warmest Quarter	mm
${\rm{BIO}}19=\mathop{\sum }\limits_{i=1}^{i=3}{{PPT}}_{i}$ where *i* ϵ ${Q}_{T{\min }}$	Precipitation of Coldest Quarter	mm

Temperature variables are expressed in °C, precipitation variables in mm, and derived indices in %. Quarterly variables are calculated from rolling three-month periods, with wettest (WQ_PPT_) and driest (DQ_PPT_) quarters identified based on total precipitation, and warmest (Q_Tmax_) and coldest (Q_Tmin_) quarters based on mean temperature. Here, i denotes month, T_max_, T_min_, and T_mean_ represent monthly mean maximum, minimum, and mean temperature, and PPT represents total monthly precipitation.

This methodological framework is transferable and could be adapted to other countries, as long as appropriate high-resolution climate data, often generated through RCMs simulations, are available. When such data exist, the framework provides a flexible tool for producing localized bioclimatic datasets to support climate impact assessments and inform regional planning efforts.

A summary of the calculation of the 19 bioclimatic variables is provided in Table 2, following the WorldClim methodology, with the corresponding symbols also described in Table 2. One key difference lies in the calculation of temperature seasonality (BIO4). WorldClim defines BIO4 as the standard deviation of monthly mean temperatures, scaled by a factor of 100 (Eq. 1):

1 $${BIO}4={SD}\left\{{{Tmean}}_{1},\ldots ,{{Tmean}}_{12}\right\}\times 100$$

In contrast, the Python BIOCLIM sub-module defines BIO4 as the coefficient of variation of monthly mean temperatures, calculated as the standard deviation divided by the annual mean temperature (BIO1) and expressed as a percentage (Eq. 2):

2 $${BIO}4=\frac{{SD}(\left\{{{Tmean}}_{1},\ldots ,{{Tmean}}_{12}\right\})}{{BIO}1}\times 100$$

To ensure compatibility and allow direct comparison with WorldClim data, we adhere to WorldClim’s definition. BIO4 was therefore first calculated using Eq. 2 and then multiplied by BIO1, thereby converting the coefficient of variation formulation to the standard deviation-based definition used by WorldClim (Eq. 1).

Although the process for generating observational and projected bioclimatic variables is similar, projected variables require additional steps. Typically, multiple input datasets lead to multiple bioclimatic datasets. To handle this, we used the glob package from the Python standard library^62^ along with Xclim’s ensemble functionality^58^, and developed a Python function to generate a single ensemble dataset (using the mean for observational data and percentiles for projections). All ensemble datasets are stored as NetCDF files (*.nc). Additionally, to facilitate further processing and compatibility with GIS platforms such as QGIS or ArcGIS, a Python function was developed to convert NetCDF files into Geotiff or GeoJSON formats.

### Future projections & percentiles

**Fig. 1:**
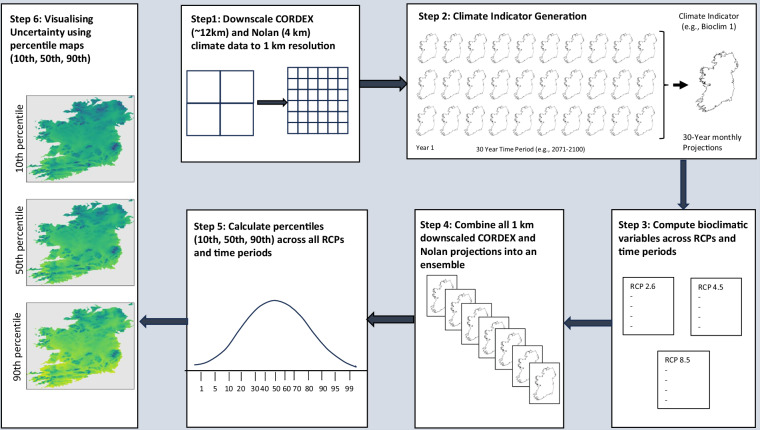
Workflow for generating projected bioclimatic variables and percentile-based ensembles in the Translate BioClim dataset. Downscaled CORDEX and Nolan projections are used to compute bioclimatic variables across Representative Concentration Pathway (RCP) scenarios and time periods, combined into an ensemble, and summarised using percentile-based outputs (10th, 50th, and 90th).

Figure 1 illustrates the process for generating projected bioclimatic variables. After producing monthly climate data at ~1 km resolution from both the CORDEX and Nolan & Flanagan (2020)^34^ ensembles, bioclimatic variables are generated for each time period (2021–2050, 2041–2070, and 2071–2100) under three RCP scenarios (RCP2.6, RCP4.5, and RCP8.5). This process results in approximately 200 climate datasets across both ensembles.

WorldClim and CHELSA offer projected bioclimatic variables using different input climate datasets from various GCMs, making it challenging for users to determine which is most appropriate for their needs. To address this, we combined all ~200 projected bioclimatic datasets using the glob package from the Python standard library^62^ along with Xclim ensemble packages^58^ to generate three percentiles (e.g., 10th, 50th, and 90th) for each variable. These percentiles summarize the distribution of projections across the ensemble and represent a range of plausible future climate conditions, from lower-end to median and higher-end estimates, helping users explore potential variability in future scenarios. In the final step, these percentiles are converted into Geotiff file format for easier use. For end-users, we also provide all individual projections sub-divided into RCPs and time periods. Percentile calculations are applied only to the ensemble of future model simulations, whereas the observational dataset represents a single historical baseline.

Percentiles (10th, 50th, and 90th) were calculated using three approaches to provide flexible options for users:

1. a full ensemble approach, in which all RCMs, RCPs, and time periods were combined to produce a single set of percentile projections for each bioclimatic variable (e.g., the 10th percentile of BIO12 representing annual precipitation aggregated across all models, scenarios, and periods).
2. a period-specific approach, which combined all RCMs and RCPs within each individual time period to produce percentiles specific to each time period (e.g., the 50th percentile of BIO12 for 2041–2070, combining all scenarios).
3. a scenario-specific approach, which computed percentiles across RCMs within each RCP–time period pair (e.g., the 90th percentile of BIO12 for RCP 4.5 during 2071–2100).

This multi-level approach allows users to select percentile datasets tailored to broad, period-specific, or scenario-specific research and decision-making needs. The full ensemble approach (Approach 1) serves as the basis for the data and analyses presented in this paper, while the other percentile datasets were developed to support future applications requiring time- or scenario-specific information.

While Approaches 1 and 2 provide simplified summaries of uncertainty across multiple scenarios and time periods, combining these projections into a single statistical distribution integrates different climate trajectories and should therefore be interpreted as a generalized representation of possible future conditions rather than the evolution of a specific scenario pathway.

Percentiles were computed from the empirical distribution of bioclimatic values across all model simulations for each grid cell. For each grid cell and variable, the relevant simulations were pooled to form a distribution of values across the ensemble. In the full ensemble approach (Approach 1), this distribution consists of 9 × n values per grid cell (three time periods × three RCP scenarios × n RCM simulations). In the period-specific approach (Approach 2), the distribution contains 3 × n values per grid cell (three RCP scenarios × n simulations). In the scenario-specific approach (Approach 3), the distribution contains n values per grid cell, corresponding to the number of available RCM simulations for each RCP–period combination. Percentiles (10th, 50th, and 90th) were then calculated from these distributions, treating all model simulations equally. The spread between the 10th and 90th percentiles therefore provide an indication of uncertainty arising from differences among model simulations.

All code used to generate both observational and future bioclimatic variables is publicly available on Zenodo^63^ to support transparency and reproducibility. The materials include an organized data and folder structure to facilitate understanding and reuse. The complete set of scripts and documentation is also publicly available on Zenodo (10.5281/zenodo.16835656).

## Data Records

The archived dataset includes the final bioclimatic outputs (3_output_BioClim), available on Zenodo^64^. The full folder structure described below (including 0_data and 2_pipeline) reflects the data processing workflow used by the accompanying code and is provided to support reproducibility.

The dataset is organized into three main directories (0_data, 2_pipeline, and 3_output_BioClim) each corresponding to a stage in the data processing workflow.

The 0_data folder contains all input climate datasets. It includes an obs subfolder with observed monthly minimum, maximum, and mean temperature, as well as precipitation data for the baseline period (1976–2005). A projections subfolder contains future climate data organized by three time periods (2021–2050, 2041–2070, 2071–2100), three RCP scenarios (2.6, 4.5, 8.5), and three sensitivity levels (low, mid, high), with each subfolder containing monthly temperature and precipitation variables.

The 2_pipeline folder stores intermediate files generated during preprocessing and transformation. Its structure mirrors that of the input data, enabling consistent data handling and facilitating the transition from raw inputs to final outputs.

The 3_output_BioClim folder contains the final set of 19 bioclimatic variables (BIO1–BIO19) in GeoTIFF (.tif) format at ~1 km resolution in the EPSG:4326 (WGS 84) coordinate system. It includes an ‘Obs’ subfolder, containing one file per variable for the observational baseline period (1976–2005), derived from a dense national station network using quality-controlled interpolation. The ‘projections’ subfolder provides future climate data organized into three complementary structures:

- full_ensemble, containing 57 files (19 variables × 3 percentiles: 10th, 50th, and 90th) aggregated across all models, scenarios, and time periods (2021–2100);
- periods, grouping projections into three time windows (2021–2050, 2041–2070, and 2071–2100), each containing 57 files;
- periods_rcps, offering the most detailed structure, organizing outputs by both time period and emissions scenario (RCP2.6, RCP4.5, RCP8.5), with three percentile-based GeoTIFFs per variable.

## Technical Validation

### Statistical comparison

To validate our datasets, we evaluated the agreement between the global climatological datasets (WorldClim and CHELSA) and Translate BioClim datasets. Both global datasets were used as benchmarks to assess the spatial consistency of the Translate-derived bioclimatic variables across Ireland. We used the highest available resolution for both datasets (~30 arc-seconds, ~1 km), allowing consistent spatial comparison with the Translate BioClim dataset^24,27^. While spatially comparable, Translate BioClim incorporates high-resolution, Ireland-specific climate inputs that enable more accurate representation of fine-scale climatic variability, features often not captured by global datasets like WorldClim or CHELSA.

To achieve the statistical comparison, we used multiple performance metrics: the coefficient of determination (R²), root mean square error (RMSE), and normalized RMSE (nRMSE). R² was applied as a similarity measure to quantify how closely the values from the Translate BioClim dataset matched those of the global climatological datasets. While R² is traditionally used to assess the accuracy of a model’s predictions (e.g., from a statistical or machine learning model) against observed data, in this context it was used to evaluate spatial similarity between two climate datasets and indicates the proportion of spatial variance shared between the two datasets for each bioclimatic variable, calculated across all grid cells over Ireland. In these comparisons, the Translate-derived bioclimatic variables were considered as the observed values, while the corresponding variables from WorldClim or CHELSA were treated as the predicted or comparison values. Mathematically, R² is defined as: ${R}^{2}=1-\frac{\sum {({y}_{i}-{\hat{y}}_{i})}^{2}}{{\sum ({y}_{i}-{\bar{y}}_{i})}^{2}}$.where:

- ${y}_{i}$ are the values from the Translate BioClim dataset (observed),
- ${\hat{y}}_{i}$ are the corresponding values from the comparison dataset, WorldClim or CHELSA (predicted/comparison), and
- ${\bar{y}}_{i}$ is the mean of the Translate BioClim values.

R² values range from negative values to 1, with values closer to 1 indicating stronger spatial similarity between the datasets. The R² reported here refers to the coefficient of determination based on the residual sum of squares, not the square of the Pearson correlation coefficient. Therefore, R² values can be negative if the comparison data fit the observations worse than using the mean of the observed data. In such cases, the difference between the Translate BioClim values and the comparison dataset exceeds the difference between the Translate BioClim values and their mean.

RMSE was used to calculate the average magnitude of error in the same units as the variable, providing a straightforward interpretation of differences. Lower RMSE values indicate smaller differences between datasets, whereas higher values suggest greater divergence. To allow comparison across variables with different scales, normalized RMSE (nRMSE) was calculated by dividing RMSE by either the range or mean of observed values. This standardization allows relative comparison of errors across variables. In addition to these metrics, the Wilcoxon signed-rank test, a non-parametric statistical test, was employed to assess whether there were statistically significant differences between paired values from the Translate BioClim dataset and each comparison dataset. This test is particularly useful to identify systematic differences when the assumptions of normality are not guaranteed. Smaller p-values (typically below 0.05) indicate significant differences between datasets. These metrics and tests were computed individually for each bioclimatic variable to capture variable-specific differences in spatial patterns and magnitudes.

### Comparison of observational bioclimatic variables across datasets

**Fig. 2:**
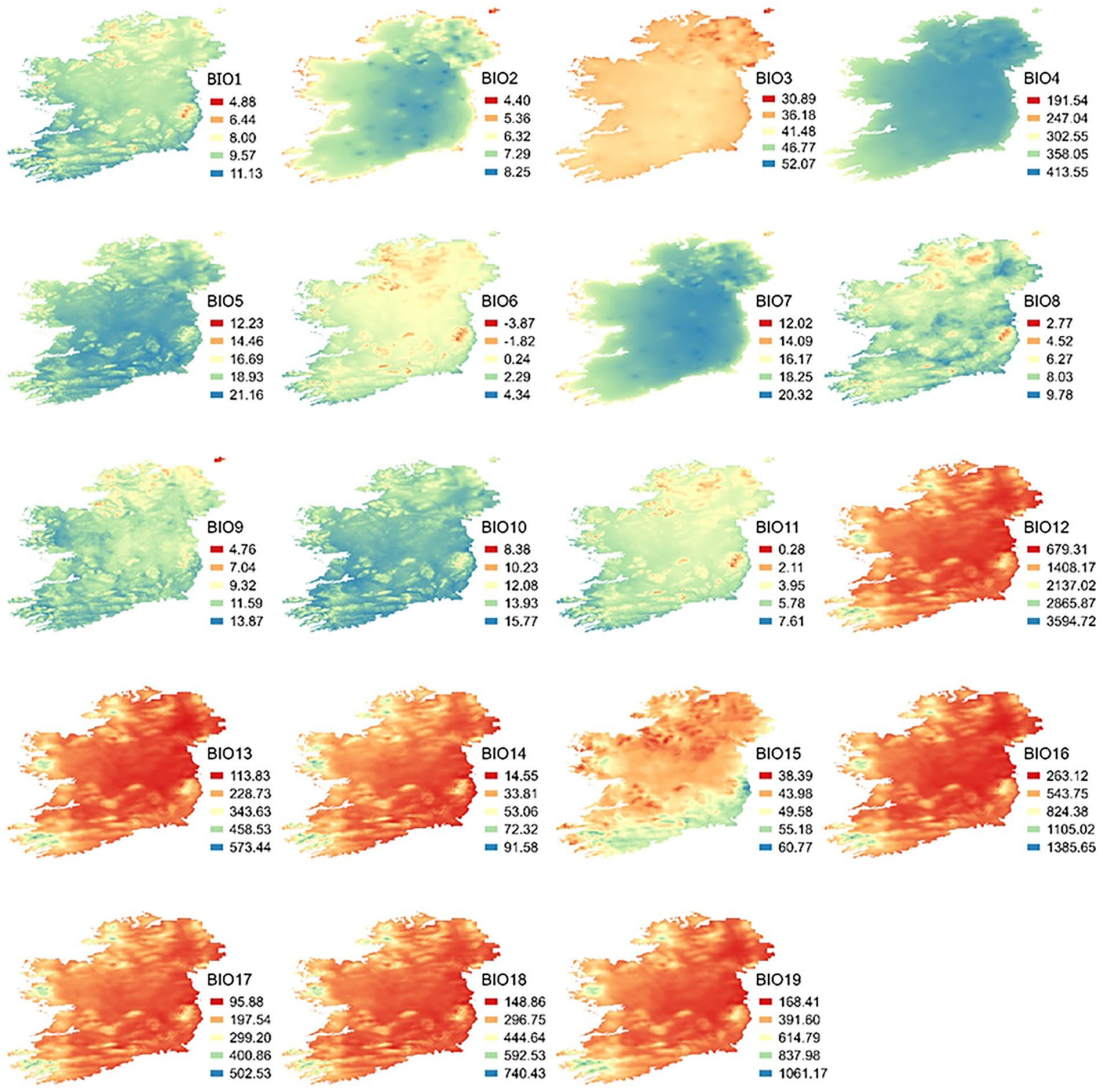
Spatial distribution of the 19 bioclimatic variables derived from the Translate project dataset for the observed reference period (1976–2005), expressed in temperature (°C), precipitation (mm).

Figure 2 illustrates all 19 high-resolution observational bioclimatic variables for Ireland, derived from the Translate BioClim dataset. To assess the reliability of these variables, we compared them with corresponding bioclimatic variables from two widely used global climatological datasets: WorldClim and CHELSA.

All three datasets provide observationally derived climate surfaces but differ in their temporal coverage, underlying data sources and modelling approaches. The Translate BioClim dataset represents the period 1976–2005 and is derived from a dense network of quality-controlled national weather stations across Ireland. In contrast, global datasets such as WorldClim^65^ (1970–2000) and CHELSA^66^ (1981–2010) are generated from more sparsely distributed global observations using spatial interpolation and statistical downscaling methods, with CHELSA additionally incorporating environmental predictors and atmospheric reanalysis data.

To enable direct comparison between datasets, all variables were normalised to a 0–1 range using the combined minimum and maximum values of the datasets being compared. Differences were then calculated between the normalised layers (Translate BioClim − comparison dataset). Consequently, the difference maps represent relative spatial differences rather than absolute differences in climatic units. Positive values indicate areas where Translate BioClim estimates are higher than the comparison dataset, while negative values indicate higher values in the comparison dataset.

### Comparison of observational bioclimatic variables: translate bioclim vs worldclim

**Fig. 3:**
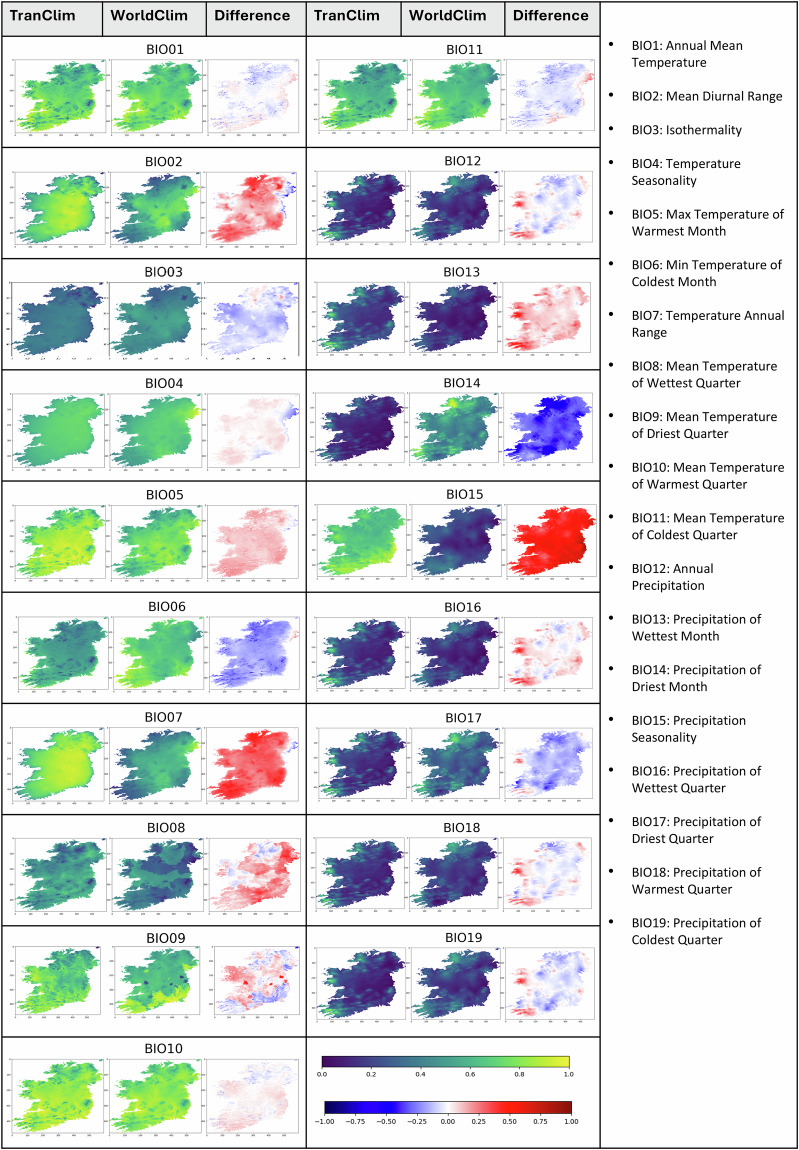
Spatial comparison of bioclimatic variables from Translate BioClim (1976–2005) and WorldClim (1970–2000). Both datasets were normalized prior to comparison. For each variable, maps show the normalized Translate dataset, the normalized WorldClim dataset, and their normalized difference (Translate − WorldClim).

The normalised Translate BioClim and WorldClim datasets show broadly similar spatial patterns across Ireland (Fig. 3), although some differences are evident. The mapped representation of differences (Fig. 3) highlights areas where changes in the values are larger between the two sources or where differences are minimal. For example, the central region of Ireland shows greater consistency between the data sources with fewer differences reported for both temperature-related and precipitation-related variables with most differences falling within approximately −0.25 to 0.25 in the normalised scale, as indicated by the colour bar.

Some differences between the datasets are expected due to variations in data sources, temporal resolution, and methodological approaches. For example, WorldClim derives mean temperature from monthly aggregated minimum and maximum temperatures^24^, whereas Translate BioClim calculates it from daily observations recorded every three hours. Although this difference likely has a limited overall impact, it may influence how temperature extremes are represented. In addition, global datasets such as WorldClim rely on interpolation of more sparsely distributed station observations across large spatial domains, which can smooth local climatic variability, particularly in regions with complex terrain such as the mountainous and western coastal areas of Ireland where topography strongly influences precipitation patterns^20^.

Some variables reflect different temporal resolutions, such as monthly, seasonal or annual trends, which may result in larger differences due to the accumulation of values over longer time periods. For example, when comparing daily precipitation between two datasets, differences between datasets at a daily scale may be relatively minor, such as a few millimetres. However, when examining annual precipitation, these small daily differences can accumulate, leading to a much larger total difference over the year. This accumulation highlights the importance of temporal resolution when interpreting and comparing climate data.

Overall, temperature-related variables (BIO1–BIO11) show strong agreement between the datasets, with relatively small and spatially smooth differences and no clear systematic bias toward either dataset. In contrast, precipitation-related variables (BIO12–BIO19) exhibit larger spatial differences, particularly for seasonal or extreme conditions. This reflects cumulative effects in precipitation, whereas temperature-related variables are based on averages (e.g., monthly or annual means) and are less sensitive to small daily variations.

Across several precipitation variables, the comparison suggests that the Translate BioClim dataset preserves stronger precipitation gradients and extremes across Ireland, particularly in western Atlantic-influenced regions, whereas the WorldClim dataset tends to produce smoother spatial patterns with comparatively higher precipitation estimates during drier periods.

**Fig. 4:**
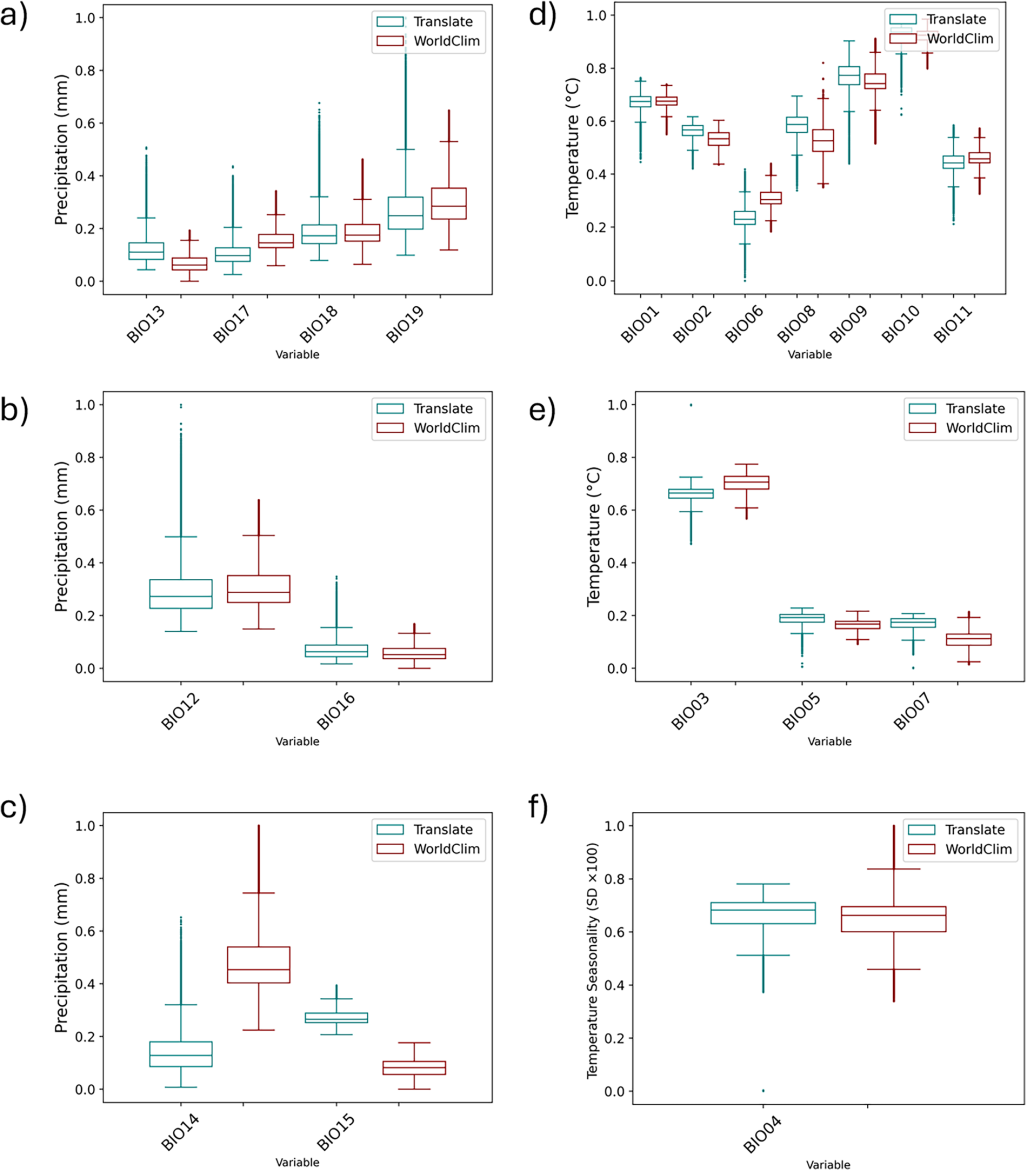
Boxplot comparison of bioclimatic variables from the TRANSLATE BioClim dataset (1976–2005) and WorldClim (1970–2000), showing the median, interquartile range (IQR), whiskers (1.5 × IQR), and outliers. Panels are grouped as follows: (**a**) precipitation of seasonal quarters (BIO13, BIO17, BIO18, BIO19); (**b**) annual precipitation and precipitation of the wettest quarter (BIO12, BIO16); (**c**) precipitation of the driest month and precipitation seasonality (BIO14, BIO15); (d) temperature-related variables including annual and seasonal mean temperatures (BIO1, BIO2, BIO6, BIO8, BIO9, BIO10, BIO11); (**e**) temperature variability and extremes (BIO3, BIO5, BIO7); and (**f**) temperature seasonality (BIO4).

Figure 4 illustrates that, in general, the Translate BioClim and WorldClim data sources exhibit similar patterns. However, while the medians and interquartile ranges are only slightly different, the main differences occur beyond the interquartile range, particularly in the extreme values.

Translate BioClim shows more lower extreme values for temperature-related variables such as BIO1, BIO2, BIO9, BIO10, and BIO11 BIO11 (annual and mean temperatures of the warmest and coldest quarters) (Fig. 4d), indicating colder conditions compared to WorldClim. However, while these values reflect increased variability in temperature, they do not necessarily indicate the occurrence of extreme cold events. Despite the similarity in interquartile range, the greater dispersion in Translate BioClim indicates more variability in colder conditions. Additional temperature-related variables (BIO3, BIO5, and BIO7) show broadly similar distributions between the two datasets (Fig. 4e), although Translate BioClim again exhibits slightly greater variability at the lower tails.

Translate BioClim also shows more extremes at the upper tails of the distributions for precipitation-related variables such as BIO13, BIO17, BIO18, and BIO19 (precipitation of seasonal quarters) (Fig. 4a), as well as BIO12, BIO16 and BIO15 (annual precipitation, precipitation of the wettest quarter and precipitation seasonality) (Fig. 4b,c). Translate BioClim exhibits lower values for BIO14 (precipitation of the driest month) compared with WorldClim. This suggests that, while the single driest month is lower in Translate BioClim, BIO17 (precipitation of the driest quarter) still shows higher upper-tail values, indicating comparatively higher precipitation during the remaining months of the driest quarter.

These higher extreme values in Translate BioClim indicate wetter and colder conditions relative to WorldClim, although they should not be interpreted as direct evidence of extreme weather and colder events. The differences reflect variability beyond the interquartile range, particularly under certain conditions such as in coastal, upland, and western regions where local climatic variation is more pronounced. This highlights how the datasets represent climatic patterns differently and provides insight into regional climate variability.

A different pattern appears for BIO4 (temperature seasonality; Fig. 4f), which represents variability in monthly mean temperatures rather than absolute temperature levels. In this case, WorldClim shows slightly greater dispersion, indicating stronger seasonal variability. This may reflect differences in how global interpolation methods represent broader spatial gradients compared with the dense local observational network underlying the Translate BioClim dataset.

Focusing on extreme values provides valuable insight into how both datasets behave under various conditions. The extreme values correspond to individual grid cells and represent valid climatic extremes captured by the high-resolution Translate BioClim dataset, rather than statistical outliers. For example, in BIO12 (annual precipitation), the highest value (around 3590 mm) occurs near Ballaghbeama Gap and Mullaghanattin in County Kerry. Historical records support this, with a maximum annual rainfall of 3964.9 mm observed at Ballaghbeama Gap in 1960 (Met Éireann)^67^, indicating these values reflect actual climatic extremes. In this area, the highest precipitation value recorded by WorldClim is 2526 mm, while Translate BioClim reports a higher value of 3033 mm. Similarly, near Croaghgorm in Donegal, where WorldClim’s highest precipitation occurs (2573 mm), Translate BioClim records a higher value (2966 mm). These differences highlight Translate BioClim’s enhanced ability to capture localized climatic variability often underrepresented in coarser datasets.

A similar check was performed for temperature extremes. The lowest daily minimum temperature in the Translate BioClim dataset (−17.66 °C) occurs on 2 January 1979 in the Lullymore area of County Kildare, closely matching the Met Éireann record of −18.8 °C observed at the Lullymore station on the same date (Met Éireann)^67^. This agreement supports the dataset’s ability to capture observed extreme temperature conditions. As daily temperature data from WorldClim were not available to us, this comparison was performed only for the Translate BioClim dataset.

Table 3 summarizes the performance metrics (R², RMSE, and nRMSE) comparing WorldClim and Translate BioClim for each bioclimatic variable. RMSE and nRMSE values varied by variable type. Among temperature-related variables, BIO06 (minimum temperature of the coldest month) and BIO07 (temperature annual range) showed the highest nRMSE values (0.73 and 0.16, respectively), reflecting greater discrepancies between the datasets. Conversely, BIO01 (annual mean temperature), BIO03 (Isothermality), BIO04 (temperature seasonality), and BIO05 (maximum temperature of the warmest month) showed relatively low nRMSE values (ranging from 0.04 to 0.06), indicating closer agreement. BIO10 (Mean Temperature of Warmest Quarter) had the lowest nRMSE (0.02), suggesting a closer match.

**Table 3:** Statistical comparison of Translate BioClim (1976–2005) and WorldClim (1970–2000) bioclimatic variables, including coefficient of determination (R²), root mean square error (RMSE), and normalized RMSE (nRMSE).

Variable	R^2^	RMSE	nRMSE
**BIO01**	0.49	0.34	0.04
**BIO02**	−0.83	0.77	0.12
**BIO03**	−1.20	2.14	0.05
**BIO04**	0.59	14.68	0.04
**BIO05**	−0.79	1.06	0.06
**BIO06**	−4.90	1.60	0.73
**BIO07**	−4.49	2.57	0.16
**BIO08**	−1.67	1.57	0.24
**BIO09**	0.04	1.12	0.10
**BIO10**	0.48	0.36	0.02
**BIO11**	0.15	0.57	0.11
**BIO12**	0.21	245.65	0.20
**BIO13**	−3.05	68.37	0.49
**BIO14**	−8.04	41.70	0.58
**BIO15**	−33.59	23.30	0.99
**BIO16**	−0.17	108.16	0.27
**BIO17**	−0.89	58.28	0.26
**BIO18**	0.25	48.89	0.19
**BIO19**	0.19	81.13	0.22

Precipitation-related variables generally exhibited larger deviations. For example, BIO15 (precipitation seasonality) had the highest nRMSE (0.99), followed by BIO14 (precipitation of driest month, nRMSE = 0.58) and BIO13 (precipitation of wettest month, nRMSE = 0.49). Even variables with a moderate relative error, such as BIO12 (annual precipitation, nRMSE = 0.20), showed very large absolute RMSE values (245.65 mm), reflecting the high magnitude of annual precipitation.

R^2^ values were generally low or negative, for example in BIO02 (−0.83), BIO06 (−4.90), and BIO15 (−33.59), indicating weak spatial agreement between the two datasets. These differences likely arise from methodological differences and variations in input data sources and time periods. As a result, even where spatial patterns appear similar, the underlying values, particularly for variables related to extremes and seasonality, can diverge significantly.

Wilcoxon signed-rank tests applied to paired values across all grid cells indicated statistically significant differences between the datasets for multiple variables (p < 0.001). These results reflect systematic differences in magnitude between the datasets. However, these differences do not necessarily imply that one dataset is superior overall. The Translate BioClim dataset exhibits a larger range of variability, reflected in wider value distributions, while overall spatial patterns between two datasets remain quite similar, especially across central Ireland (Figs. 3, 4).

Previous studies have reported strong spatial agreement between remote sensing datasets and WorldClim, particularly in overall patterns. Amiri (2020)^68^ found that most remote sensing-derived bioclimatic variables closely matched WorldClim in terms of spatial patterns and correlations, except for variables involving temperature extremes. Similarly, a study on Mongolia^69^ showed strong spatial agreement between satellite-based estimates and WorldClim. These findings indicate that remote sensing data generally reinforce WorldClim’s representation of climate patterns. In contrast, our results show that the Translate BioClim dataset diverges from WorldClim in the magnitude and variability of certain variables. Differences are particularly evident for precipitation-related variables (Fig. 4a–c), while temperature-related variables generally show closer agreement (Fig. 4d,e).

While remote sensing often underestimates extremes^68^, Translate BioClim’s high-frequency observations may more accurately capture colder conditions, as reflected in the lower extremes in our results. Although statistical differences were observed, especially in the magnitude of values, the broad spatial trends remained consistent. This indicates that, whereas remote sensing aligns closely with global datasets like WorldClim, regionally refined products such as Translate BioClim can add value by capturing finer-scale climate variation crucial for local studies and regional climate analyses.

### Comparison of observational bioclimatic variables: translate bioclim vs CHELSA

**Fig. 5:**
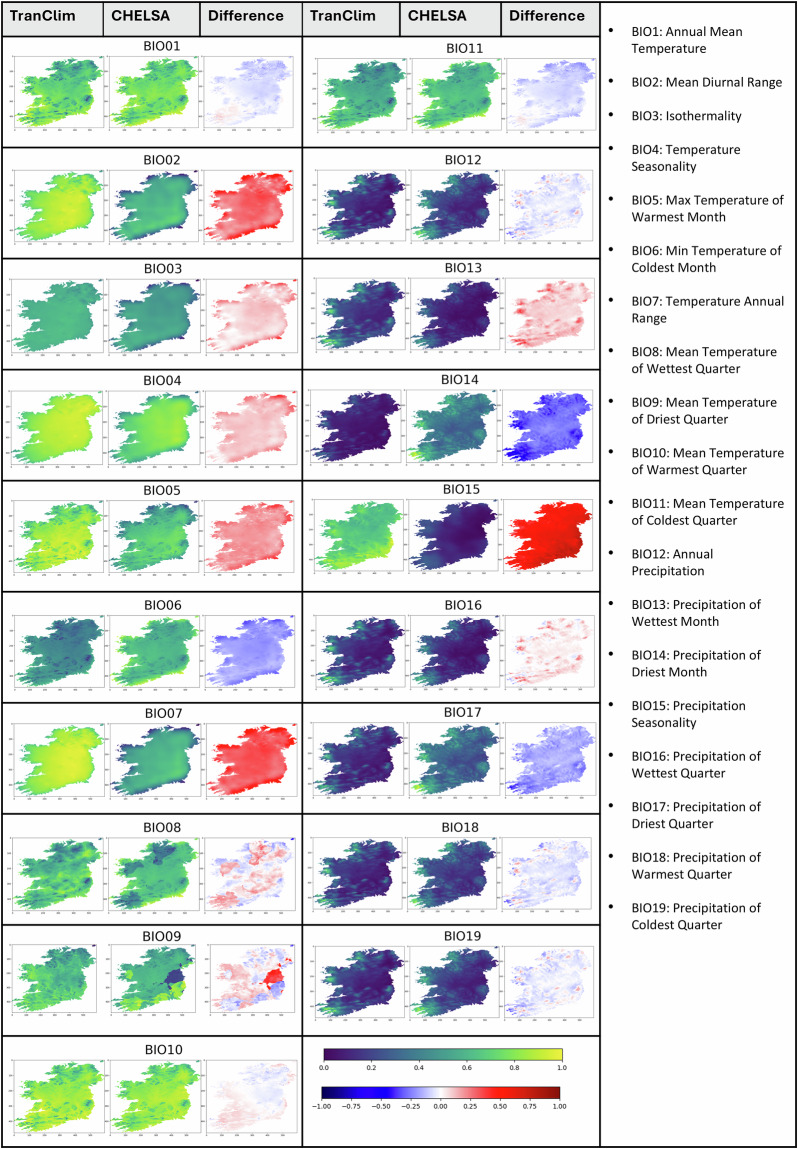
Spatial comparison of bioclimatic variables from Translate BioClim (1976–2005) and CHELSA (1981–2010). Both datasets were normalized prior to comparison. For each variable, maps show the normalized Translate dataset, the normalized CHELSA dataset, and their normalized differences (Translate BioClim − CHELSA BioClim).

To further evaluate the consistency of the Translate BioClim dataset, the observational bioclimatic variables were also compared with the CHELSA observational dataset (Fig. 5). Overall, the two datasets show broadly similar spatial patterns across Ireland and reproduce the main climatic gradients associated with Atlantic influence and topographic variability.

For temperature-related variables (BIO1–BIO11), the spatial distributions are highly consistent between the two datasets. Mean temperature variables such as BIO1 (annual mean temperature) and BIO10 (mean temperature of the warmest quarter) show similar spatial gradients, with the Translate dataset tending to be relatively cooler than CHELSA in northern regions and closer to or slightly warmer than CHELSA in southern and coastal areas. In contrast, BIO5 (maximum temperature of the warmest month) exhibits a different pattern, with the Translate dataset generally showing higher values than CHELSA across most of Ireland and less pronounced spatial variation. Some differences are evident for winter-related variables such as BIO6 (minimum temperature of the coldest month) and BIO11 (mean temperature of the coldest quarter), where the Translate dataset tends to represent slightly colder conditions across Ireland, with more pronounced differences in northern areas. Variables such as BIO7 (temperature annual range) also indicate slightly higher values in the Translate dataset, reflecting stronger seasonal contrasts.

For precipitation-related variables (BIO12–BIO19), both datasets capture the characteristic west–east precipitation gradient across Ireland, with higher precipitation in western and southwestern regions and lower values toward the east. For example, BIO12 (annual precipitation) and BIO16 (precipitation of the wettest quarter) show very similar large-scale spatial patterns in both datasets. However, localized differences occur in mountainous and Atlantic-exposed regions, reflecting differences in how precipitation is represented in areas strongly influenced by topography. Variables associated with wetter conditions, such as BIO13 (precipitation of the wettest month), often show slightly higher values in the Translate dataset, whereas CHELSA tends to produce higher precipitation estimates during the driest periods, as reflected in BIO14 (precipitation of the driest month) and BIO17 (precipitation of the driest quarter). BIO15 (precipitation seasonality) also shows higher values in the Translate dataset, suggesting stronger seasonal contrasts in precipitation.

Overall, the comparison indicates strong agreement between the Translate BioClim and CHELSA datasets, with both reproducing the main climatic gradients across Ireland. The observed differences are generally localized and likely reflect methodological differences in spatial interpolation, statistical downscaling, and the treatment of topographic influences on precipitation.

The statistical comparison generally supports the spatial patterns observed in the maps. Several temperature variables, such as BIO01 (annual mean temperature) and BIO10 (mean temperature of the warmest quarter), show moderate to strong agreement between the datasets (R² = 0.43 and 0.71, respectively), with relatively low nRMSE values (Table 4). However, variables associated with winter temperature conditions and temperature variability, such as BIO06 (minimum temperature of the coldest month) and BIO07 (temperature annual range), show larger deviations between the datasets, reflected in higher nRMSE values and localized differences visible in the spatial maps. For precipitation variables, strong agreement is observed for large-scale indicators such as BIO12 (annual precipitation), BIO16 (precipitation of the wettest quarter), and BIO19 (precipitation of the coldest quarter), which show relatively high R² values (0.66–0.77). In contrast, variables related to precipitation seasonality and dry conditions, particularly BIO15 and BIO14, exhibit larger errors, indicating greater differences between the datasets in representing seasonal precipitation variability and dry-period precipitation (Table 4). Statistical testing using the Wilcoxon signed-rank test confirmed significant differences between the Translate BioClim and CHELSA datasets across multiple variables (p < 0.001).

**Table 4:** Statistical comparison of Translate BioClim (1976–2005) and CHELSA BioClim (1981–2010) bioclimatic variables, including coefficient of determination (R^2^), root mean square error (RMSE), and normalized RMSE (nRMSE).

Variable	R^2^	RMSE	nRMSE
**BIO01**	0.43	0.41	0.04
**BIO02**	−4.90	1.90	0.36
**BIO03**	−1.11	3.92	0.11
**BIO04**	−0.72	28.81	0.08
**BIO05**	−2.73	1.82	0.10
**BIO06**	−4.24	1.98	0.74
**BIO07**	−6.79	3.77	0.25
**BIO08**	0.39	0.78	0.11
**BIO09**	0.11	1.46	0.13
**BIO10**	0.71	0.31	0.02
**BIO11**	−0.003	0.66	0.12
**BIO12**	0.74	171.34	0.13
**BIO13**	−0.90	55.99	0.39
**BIO14**	−7.76	51.34	0.63
**BIO15**	−61.64	27.76	1.47
**BIO16**	0.66	69.30	0.17
**BIO17**	−1.60	82.68	0.32
**BIO18**	0.58	40.38	0.14
**BIO19**	0.77	55.06	0.15

### Comparison of future bioclimatic projections

Both WorldClim and CHELSA provide future climate projections based on statistically downscaled outputs from multiple GCMs. In their most recent versions, these datasets primarily provide CMIP6 projections under Shared Socio-economic Pathways (SSPs), although CMIP5 projections based on Representative Concentration Pathways (RCPs) are also available. However, these CMIP5 projections are not consistently available for all time periods across datasets. The data are organized as individual files for each combination of GCM, scenario, and 20-year period (e.g., 2021–2040, 2041–2060, 2061–2080, 2081–2100). This structure results in a collection of files for each variable, making it challenging for non-expert users to select the most appropriate dataset for their specific needs. Although percentiles could, in principle, be calculated across GCM runs within each scenario and time period, these datasets do not provide such aggregated summaries directly.

In contrast, the Translate BioClim dataset provides ensemble-based projections summarised as percentiles (e.g., 10th, 50th, and 90th), for managing uncertainty in bioclimatic modelling. Therefore, Translate BioClim facilitates easier selection of datasets that align with users’ specific needs, whether they pertain to low, median, or more extreme climate scenarios.

In this study, future projections from the Translate BioClim dataset were first compared with the historical Translate BioClim baseline using the full ensemble percentile dataset to assess projected climate changes relative to historical baseline conditions. To further evaluate these projections against other widely used datasets, additional comparisons were conducted with WorldClim and CHELSA. However, direct comparisons between aggregated percentile-based datasets and individual-model projections are not methodologically straightforward because of differences in dataset structure and underlying modelling frameworks. For consistency, the comparison was therefore restricted to a common scenario and time period available across datasets (RCP4.5 for 2041–2060). Percentiles (10th, 50th, and 90th) were calculated across the available model simulations for WorldClim^70^ and CHELSA^71^ and compared with the corresponding Translate BioClim projections (RCP4.5 for 2041–2070, representing the closest available mid-century period).

### Comparison of translate future projections vs observations

**Fig. 6:**
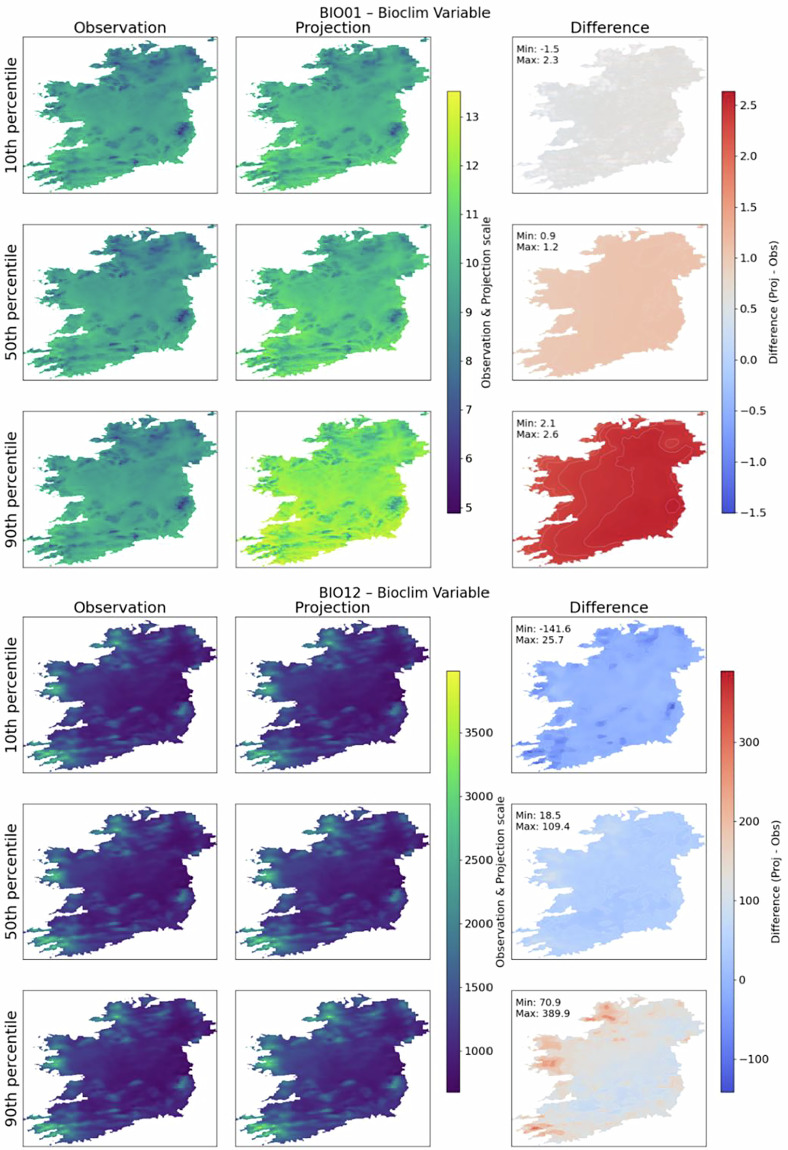
Comparison of the observational (historical) and projected Translate BioClim variables. Historical values for the reference period (1976–2005) are compared with projected values derived from the full ensemble, from which the 10th, 50th, and 90th percentiles were calculated. Maps show BIO01 (annual mean temperature, °C) and BIO12 (annual precipitation, mm). For each variable, rows show the historical dataset alongside each of the projected percentiles (10th, 50th, and 90th), while columns show the historical values, the projected values, and their difference (projected −historical).

Figure 6 compares the projected values of two bioclimatic variables, BIO01 (Annual Mean Temperature) and BIO12 (Annual Precipitation), at the 10th, 50th, and 90th percentiles with their historical values. This highlights how these climate variables are expected to change in the future compared to the past. For BIO01, the projected increase relative to the historical baseline is positive everywhere, with little variation across the country, reflecting how the global climate change signal has been separated from the climate influence of localized geographic features. Changes are slightly greater in the eastern regions, while the western and southern areas show smaller changes across all three percentiles, although that geographical variability is swamped by the inter-model variability (as represented by the different percentiles) and so are not obvious in the common shading scheme. In terms of range, the 10th percentile differences range from 0.45 °C to 0.75 °C, the 50th percentile differences range from 0.95 °C to 1.20 °C, and the 90th percentile differences range from 2.1 °C to 2.8 °C. These values reflect both spatial variability in warming across the country and the spread of model outputs within the ensemble, illustrating the range of possible climate futures.

For BIO12 (Annual Precipitation), the differences range from −125 mm to 20 mm in the 10th percentile. In some areas, mostly along the coastal edges and away from central regions, projected precipitation is lower than historical. However, in most regions, it is slightly higher, with increases of around 20 mm. In the 50th percentile, increases range from 20 mm to 100 mm, with the eastern regions showing smaller increases (mostly under 50 mm), while other areas, particularly in the west, experience increases nearing 100 mm. In the 90th percentile, projected precipitation increases range from 100 mm to 350 mm, with the greatest increases, often exceeding 200 mm, occurring in the western, southwestern, and northwestern regions. In contrast, the eastern and central areas generally show smaller increases, around 100 to 200 mm. As with temperature, this reflects both regional climate variation and differences among model projections. Importantly, these results concur with national and regional climate projections for Ireland, which also indicate a future trend toward warmer and wetter conditions.

The comparisons between BIO01 and BIO12 show contrasting regional patterns in projected changes. While projected temperature increases (BIO01) are more pronounced in the eastern and central regions, precipitation increases (BIO12) are greater in the west, southwest, and northwest. This contrast suggests that regions with smaller temperature increases may still experience substantial increases in precipitation. These differences are most evident at the 90th percentile, moderate at the 50th, and least noticeable at the 10th percentile, highlighting how projected changes vary not only by variable and region but also across the range of future scenarios. Understanding these spatial and percentile-based differences is crucial for anticipating future climate impacts and tailoring regional climate adaptation strategies.

These trends align with the findings of Sobh (2022)^72^, who analysed CMIP6-based projections for Southeast Asia and reported notable increases in annual and seasonal precipitation, especially under high-emission scenarios. Both studies emphasize the importance of regional detail and percentile-based assessments to support climate adaptation planning. The use of percentiles in our approach, much like the multi-model means in CMIP6 analyses, enables decision-makers to choose projections that align with different levels of climate risk tolerance.

Technical validation identifies that the Translate BioClim dataset offers higher spatial detail in climate variables, and through the percentile-based projections, users can better assess uncertainty and tailor results to specific regional or sectoral applications. The ability to explore a range of scenarios provides a valuable tool for decision-makers, especially when planning for climate-sensitive sectors such as agriculture or biodiversity.

### Comparison of translate bioclim and worldclim future projections

**Fig. 7:**
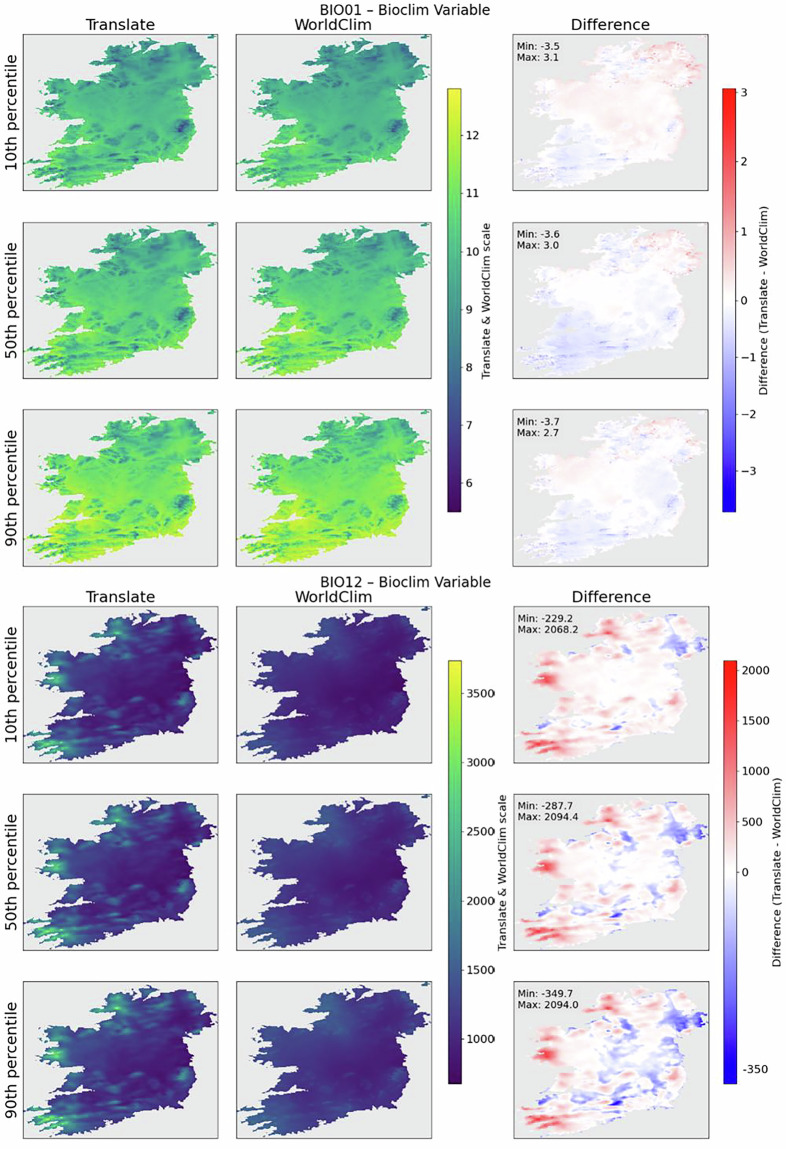
Comparison of Translate BioClim and WorldClim projections for RCP4.5. Translate projections for the period 2041–2070, derived from 29 regional climate model (RCM) simulations, were combined to obtain the 10th, 50th, and 90th percentiles of the bioclimatic variables. These are compared with WorldClim projections for the period 2041–2060, derived from 19 global climate model (GCM) simulations, from which the 10th, 50th, and 90th percentiles were similarly calculated. The figure shows maps of BIO01 (Annual Mean Temperature, °C) and BIO12 (Annual Precipitation, mm). For each variable, rows correspond to the 10th, 50th, and 90th percentiles, while columns show the Translate projection, the WorldClim projection, and the difference between the two datasets (Translate − WorldClim).

Figure 7 compares the percentile-based projections of the Translate BioClim dataset with those derived from WorldClim for BIO01 (Annual Mean Temperature) and BIO12 (Annual Precipitation). For both variables, the spatial patterns produced by the two datasets are broadly consistent, reflecting similar large-scale climatic gradients across Ireland.

For BIO01, both datasets show a clear west–east temperature gradient, with generally cooler conditions in the western and northern regions and warmer conditions in the south and southeast. The spatial distribution of temperatures is highly comparable across the 10th, 50th, and 90th percentiles. The difference maps indicate relatively small deviations between the datasets, with most differences within ±1 °C, while a few localized areas exhibit larger deviations. Slightly higher temperatures in the Translate dataset are observed in some eastern and northeastern regions, while WorldClim shows marginally higher values in parts of the southwest. Overall, however, the differences are spatially limited and do not substantially alter the overall temperature pattern across the country.

For BIO12, both datasets capture the well-known precipitation gradient across Ireland, with higher precipitation levels in the western and southwestern regions and lower values in the east and southeast. The general spatial structure of precipitation is therefore consistent between the datasets across all three percentiles. However, the difference maps show somewhat larger deviations than for temperature. In several western and coastal regions, Translate estimates higher precipitation values than WorldClim, while localized areas of lower precipitation appear in parts of the east and central regions. These differences likely reflect variations in downscaling methodology, spatial resolution, and the representation of orographic effects between the datasets.

These differences likely arise from methodological differences between the datasets, including variations in downscaling approaches, input data sources, and time periods. As a result, even when spatial patterns appear similar, the underlying values, especially for variables related to extremes and seasonality can diverge significantly.

Despite these localized differences, the overall spatial patterns of temperature and precipitation remain highly comparable between the Translate BioClim and WorldClim projections.

### Comparison of translate bioclim and CHELSA future projections

**Fig. 8:**
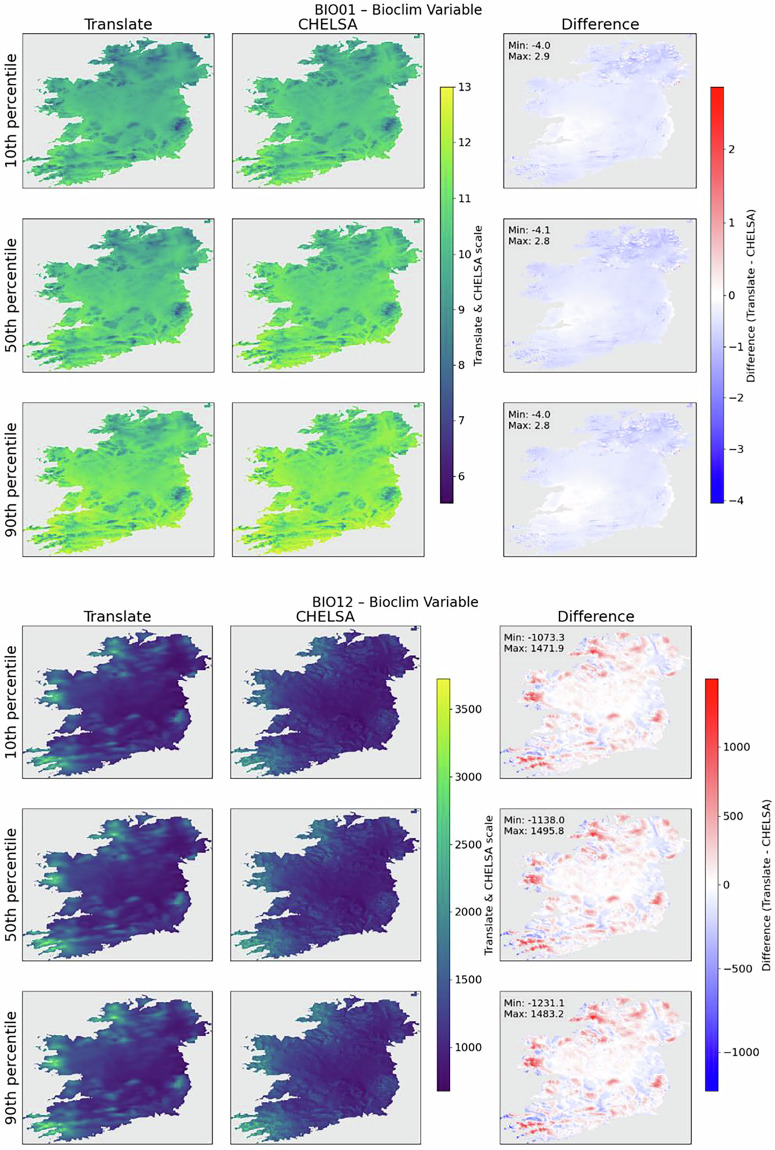
Comparison of Translate BioClim and CHELSA BioClim projections for RCP4.5. Translate projections for the period 2041–2070, derived from 29 regional climate model (RCM) simulations, were combined to obtain the 10th, 50th, and 90th percentiles of the bioclimatic variables. These are compared with CHELSA projections for the period 2041–2060, derived from 32 global climate model (GCM) simulations, from which the 10th, 50th, and 90th percentiles were similarly calculated. The figure shows maps of BIO01 (Annual Mean Temperature, °C) and BIO12 (Annual Precipitation, mm). For each variable, rows correspond to the 10th, 50th, and 90th percentiles, while columns show the Translate projection, the WorldClim projection, and the difference between the two datasets (Translate − CHELSA).

Figure 8 compares the percentile-based projections of the Translate BioClim dataset with those derived from CHELSA for BIO01 (Annual Mean Temperature) and BIO12 (Annual Precipitation). As with the WorldClim comparison, the spatial patterns of both variables are broadly consistent, capturing the main climatic gradients across Ireland.

For BIO01, both datasets show highly similar spatial temperature patterns across Ireland, with cooler conditions in the western and northern regions and warmer conditions in the south and southeast. Differences between the datasets are generally small, with most areas showing slightly negative values (around −1 to 0 °C), indicating that CHELSA estimates marginally higher temperatures than Translate in many locations, while a few localized regions exhibit larger positive or negative deviations.

For BIO12, both datasets reproduce the well-known west–east precipitation gradient across Ireland. However, the difference maps indicate somewhat larger local deviations than for temperature. In several western and coastal regions, Translate projects higher precipitation than CHELSA, while localized areas of lower precipitation appear in parts of the east and central regions.

These differences likely reflect methodological distinctions in downscaling approaches and the treatment of topographic effects. Despite these localized differences, the overall spatial patterns of temperature and precipitation remain broadly consistent between the two datasets.

## Usage Notes

The Translate BioClim dataset provides high-resolution bioclimatic variables for Ireland, including both observational data and future projections generated through a flexible percentile-based ensemble framework. Observational variables are derived from a dense network of national weather stations, ensuring accurate representation of local climate patterns, particularly under colder and wetter conditions. Compared with WorldClim, Translate BioClim offers improved spatial detail and precision due to quality-controlled interpolation at ~1 km resolution.

Future projections are based on a flexible percentile-based ensemble approach (Fig. 1), combining dynamically downscaled climate data from CMIP5 using RCMs, along with detrending, bias correction, and high-resolution modelling. Percentile-based outputs (10th, 50th, and 90th) provide a practical framework for representing uncertainty across different temporal and scenario-based scales. Users can access full ensemble projections covering 2021–2100, as well as percentiles for individual time periods and RCP scenarios. While WorldClim currently incorporates CMIP6-SSP scenarios, the Translate dataset is being updated to include CMIP6-driven RCMs^40^, enhancing its comparability and relevance for future climate assessments. Preliminary comparisons suggest that while some quantitative differences may arise, overall spatial patterns remain broadly consistent^57^.

The percentile-based ensemble projection framework offers a practical and transferable tool for managing uncertainty and supporting adaptation planning, not only in Ireland but also in other regions seeking fine-scale climate insights.

## Data Availability

The final output dataset (TIFF files) is available in a separate Zenodo^64^ record. The dataset includes the derived bioclimatic variables generated in this study. Both, Zenodo records will be made publicly accessible upon publication.

## Code availability

The complete set of Python scripts, Jupyter Notebook workflows, and folder structure documentation developed for processing climate data and generating the 19 standard bioclimatic variables for Ireland are available on Zenodo^63^. The code was developed and tested in Python 3.10.14, using the main package Xclim (0.51.0). All parameters and variables required to reproduce the results are included as examples within the code.
